# Facile Synthesis of Fe-Doped, Algae Residue-Derived Carbon Aerogels for Electrochemical Dopamine Biosensors

**DOI:** 10.3390/s24092787

**Published:** 2024-04-27

**Authors:** Hao Wu, Qin Wen, Xin Luan, Weiwei Yang, Lei Guo, Gang Wei

**Affiliations:** 1 Institute of Biomedical Engineering, College of Life Science, Qingdao University, Qingdao 266071, China; w69699169@163.com (H.W.); qwen@qdu.edu.cn (Q.W.); y962185626@126.com (W.Y.); 2 College of Chemistry and Chemical Engineering, Qingdao University, Qingdao 266071, China; luanxin0924@outlook.com

**Keywords:** algae residue, carbon aerogel, electrochemical biosensor, dopamine, iron doping

## Abstract

An abnormal level of dopamine (DA), a kind of neurotransmitter, correlates with a series of diseases, including Parkinson’s disease, Willis-Ekbom disease, attention deficit hyperactivity disorder, and schizophrenia. Hence, it is imperative to achieve a precise, rapid detection method in clinical medicine. In this study, we synthesized nanocomposite carbon aerogels (CAs) doped with iron and iron carbide, based on algae residue-derived biomass materials, using Fe(NO_3_)_3_ as the iron source. The modified glassy carbon electrode (GCE) for DA detection, denoted as CAs-Fe/GCE, was prepared through surface modification with this composite material. X-ray photoelectron spectroscopy and X-ray diffraction characterization confirmed the successful doping of iron into the as-prepared CAs. Additionally, the electrochemical behavior of DA on the modified electrode surface was investigated and the results demonstrate that the addition of the CAs-Fe promoted the electron transfer rate, thereby enhancing their sensing performance. The fabricated electrochemical DA biosensor exhibits an accurate detection of DA in the concentration within the range of 0.01~200 µM, with a detection limit of 0.0033 µM. Furthermore, the proposed biosensor is validated in real samples, showing its high applicability for the detection of DA in beverages.

## 1. Introduction

Dopamine (DA, 3,4-dihydroxyphenethylamine), a catecholamine neurotransmitter, plays a crucial role in mammalian neuroregulation [1,2]. In recent years, more and more investigations have been focused on the relationship between DA and neuropsychiatric disorders and, in clinical diagnosis, the amount of DA in human body fluids has been used as an important diagnostic indicator for Alzheimer’s disease, depression, drug addiction, schizophrenia, and Parkinson’s disease [3]. Hence, the detection and quantification of DA plays a vital role in diagnosing and preventing the aforementioned diseases [4]. Currently, the most commonly used methods for DA analysis and detection include liquid chromatography-MS [5], gas chromatography-MS [6], fluorescence sensors [7], surface plasmon resonance [8], and others. These methods, however, suffer from such drawbacks as low sensitivity, poor selectivity, and time-consuming procedures [9]. In comparison, electrochemical detection offers many advantages such as simplicity, real-time monitoring, rapid response, high sensitivity, and good selectivity, making it an ideal method for DA detection [10]. The application of excellent active materials to modify electrodes is of significance for enhancing the sensitivity and selectivity of electrochemical DA detection [11]. Therefore, the development of electrode materials with an excellent activity is crucial for the fabrication of electrochemical DA sensors [12].

Carbon-based materials have been proven to be one of the most effective candidates for fabricating electrochemical sensors. Puthongkham et al. [13] employed the electrodeposition method to modify carbon nanohorns (CNHs) onto carbon fiber microelectrodes (CFMEs), following NaOH oxidation etching, resulting in ox-CNH/CFME, which greatly enhanced the defect sites and surface oxidation groups of carbon materials and exhibited high activity in the electrochemical detection of DA. Utilizing carbon black nanoparticles (CBNs) to modify glassy carbon electrodes (GCEs), Jiang et al. [14] prepared a modified electrode (CBN/GCE) with a lower detection limit for DA and satisfactory recovery rates were observed in the presence of both DA and uric acid, indicating its high specificity for DA detection. Rana et al. [15] synthesized the carbon-based nanomaterial CGC-500 from *Parthenium hysterophorus* L. via hydrothermal-assisted carbonization; the modified electrode exhibited ultra-low detection limits and a good reproducibility and repeatability in the electrochemical detection of DA.

On the other hand, the development of new materials with a higher electrochemical activity through chemical modification and mixing with other materials has become a major focus of research [12]. Metallic materials, including noble metal nanoparticles [16] and metal oxide nanocomposites [17] have been explored. For example, Zhang et al. [18] synthesized an Fe_2_O_3_/a-G-PI-based carbon aerogel (CA) for DA detection, by modifying Fe_2_O_3_ nanoparticles onto porous Cas, using KOH-activated graphene-polyimide aerogels as the raw material. The as-synthesized catalytic material exhibited a high specific surface area and rich mesoporous and large pores, providing ample space for the in situ growth of Fe_2_O_3_ nanoparticles and effectively preventing their aggregation. In the electrochemical detection of DA, the material showed a low detection limit and a good selectivity. Additionally, Yang et al. [19] synthesized a novel Fe-doped composite (Fe/FeC@CNT) via microwave-assisted synthesis, using ferrocene and carbon nanotubes (CNTs), demonstrating good reproducibility, repeatability, and stability in the electrochemical detection of DA. Anshori et al. [20] functionalized multi-walled carbon nanotubes (MWCNTs) with silver nanoparticles, to obtain a highly sensitive and selective DA detection material. As for the detection of other biomolecules, a unique material of graphdiyne-chelated AuNPs (GDY@AuNPs) was designed and developed to achieve high-performance electrochemical sensing of Tyr [21]. Additionally, Ferlazzo et al. [22] reported the first example of a novel potentiometric sensor for the real-time monitoring of phenylalanine (Phe). The developed biosensors displayed a wide range of detection (0–5000 μM).

**Scheme 1:**
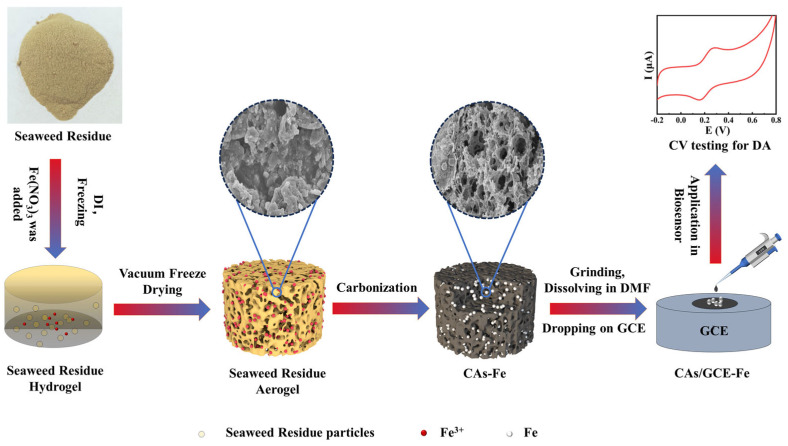
Schematic synthesis process of CAs-Fe, using AR and Fe(NO_3_)_3_ as the carbon and Fe resources, as well as the fabrication of CA-Fe-based electrochemical DA biosensors.

Therefore, we consider that employing metallic or metal oxide nanoparticles loaded onto carbon materials is an ideal approach for synthesizing catalysts with excellent electrochemical activity [23]. Algae residue (AR), as a form of biomass waste, mainly consists of crude protein and crude fiber. Compared with other waste materials such as household waste, AR possesses such characteristics as easy availability, low cost, good biocompatibility, and environmental friendliness. Currently, AR has become a popular material for synthesizing activated carbon and holds a high potential for various uses. As shown in Scheme 1, a novel Fe-doped carbon nanomaterial (CAs-Fe) is synthesized using AR as the carbon source and Fe(NO_3_)_3_ as the Fe source, via freeze-drying and carbonization processes. Then, the obtained CAs-Fe is cast onto a GCE, producing a modified electrode for the further electronic detection of DA. The obtained results indicate that, compared with non-doped CAs, CAs-Fe exhibit a higher sensitivity and a lower detection limit for DA detection. Even in the wide range of detection limits and in the presence of other interfering substances, the CAs-Fe still demonstrate excellent electrocatalytic performance, revealing enormous potential application in the electrochemical detection of DA. This study provides a feasible pathway for the high-value utilization of AR as a biomass resource and for the electrochemical detection of DA.

## 2. Materials and Methods

### 2.1. Reagents and Chemicals

The seaweed was sourced from South American brown algae, while sodium dihydrogen phosphate (NaH_2_PO_4_) and disodium hydrogen phosphate (Na_2_HPO_4_) were purchased from Sigma-Aldrich. DA was obtained from Merck. Fe(NO_3_)_3_, N,N-dimethylformamide (DMF), Nafion reagent, anhydrous ethanol, acetic acid, chitosan, and potassium nitrate (KNO_3_) were provided by China National Pharmaceutical Group Corporation. Potassium chloride, potassium ferricyanide, potassium ferrocyanide, glucose (GLU), uric acid (UA), and ascorbic acid (AA) were supplied by Shanghai Sango Biotech Co., Ltd., Shanghai, China. Collected urine samples were immediately sealed and were stored in a refrigerator. A phosphate-buffered saline (PBS) solution, of 0.1 M and pH = 7.0, was prepared by mixing NaH_2_PO_4_ and Na_2_HPO_4_ stock solutions. Deionized water with a resistivity of 18 M/cm was obtained from a Millipore Milli-Q water system (Millipore Inc., Burlington, MA, USA) and used throughout the experiment. All chemicals were of analytical grade and, unless otherwise stated, the chemicals or solvents used in this study did not require further purification.

### 2.2. Synthesis of CAs-Fe

The preparation of CAs involved freeze-drying and carbonization processes [24]. The preparation of the CAs-Fe was improved upon this basis. As illustrated in Scheme 1, South American brown algae were utilized as the raw material, which underwent processes such as rinsing, crushing, alkali dissolution, grinding, and foam filtration to obtain seaweed filter residue for the preparation of CAs-Fe. In brief, 1.5 g of seaweed residue was added to 75 mL of deionized water, followed by the addition of 1.5 g of Fe(NO_3_)_3_. The mixture was continuously stirred with a glass rod to ensure complete dissolution of the added Fe(NO_3_)_3_, resulting in a seaweed residue mixture solution. To this mixture, 0.75 g of guar gum and 7.5 mL of 1% sodium tetraborate solution were added and the mixture was frozen at −80 °C for 12 h. After freezing, the samples were vacuum-dried for 10 h in a vacuum freeze-dryer. The dried samples were then placed into a tube furnace, purged with N_2_, and heated to 700 or 1000 °C at a rate of 5 °C/min, followed by holding for 2 h and cooling to room temperature, yielding Fe-doped Cas, denoted as CAs-Fe-700 and CAs-Fe-1000, respectively [25].

### 2.3. Synthesis of Modified Electrodes

The obtained CAs, CAs-Fe-700 and CAs-Fe-1000, were ground into powders, which were then dispersed separately into DMF solution (10 mg/mL). Nafion reagent was added to the DMF solution (Nafion:DMF = 1:40) and the mixture was sonicated for 15 min, to obtain a uniform dark-black suspension. Before electrode modification, GCEs were polished on microcloth, using alumina slurries with particle sizes of 1, 0.3, and 0.05 μm, followed by rinsing with deionized water. Subsequently, the electrodes were sonicated in anhydrous ethanol and deionized water for 3 min each. Then, the CAs or CA-Fe suspension in DMF (7.5 μL) was cast onto the GCE and dried under an infrared lamp, resulting in modified electrodes that are denoted as CAs/GCE, CAs/GCE-Fe-700, and CAs/GCE-Fe-1000, respectively [26]. The prepared modified electrodes had a diameter (D) of 0.2 mm and an effective working area (S) of 3.14 × 10^−2^ cm^2^.

### 2.4. Characterization Techniques

Scanning electron microscopy (SEM) images of the samples were recorded using the Czech Tescan MIRA LMS field emission scanning electron microscope system (Tescan Company, Brno, Czech Republic). X-ray photoelectron spectroscopy (XPS) characterization of the samples was conducted on a PHI 5000 Versa Probe III spectrometer (ULVAC PHI Company, Kanagawa, Japan), with energy calibration, using the C 1s signal at 284.8 eV. Raman spectra were obtained using a Renishaw Via 1000 Raman spectrometer(Renishaw company, Gloucestershire, UK). X-ray diffraction (XRD) measurements were performed using a Bruker Axs diffractometer (Bruker company, Saarbrucken, Germany) with Cu Kα radiation at 40 kV and 30 mA and the scanning rate was 0.01° s^−1^ (λ = 0.154 nm). All experiments were conducted at room temperature.

### 2.5. Electrochemical Detection

All electrochemical measurements were conducted using a CHI660C electrochemical workstation (CH Instrument, Shanghai Chenhua Co., Ltd., Shanghai, China). Cyclic voltammetry (CV) was performed using a conventional three-electrode system, where a GCE or modified electrode served as the working electrode, an Ag/AgCl electrode functioned as the reference electrode, and a platinum foil electrode was employed as the counter electrode.

## 3. Results and Discussion

### 3.1. Characterization of CAs-Fe

**Figure 1:**
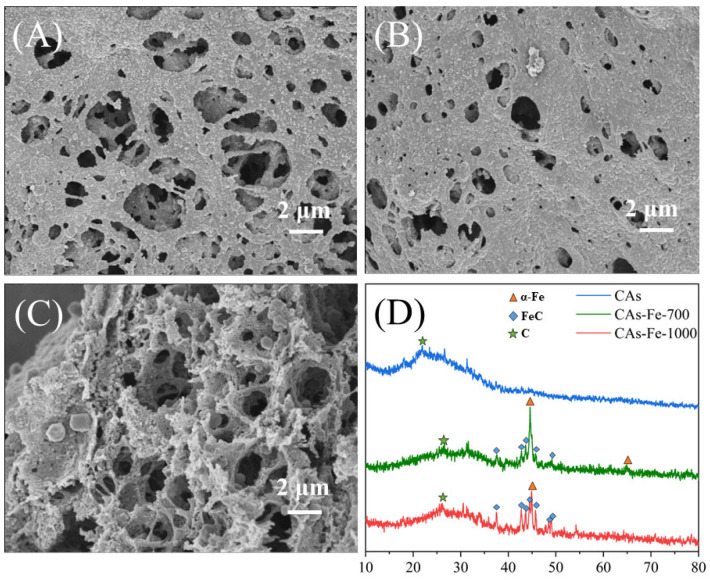
SEM images of (**A**) CAs, (**B**) CAs-Fe-700, and (**C**) CAs-Fe-1000. (**D**) XRD spectra of CAs, CAs-Fe-700, and CAs-Fe-1000.

As depicted in Figure 1A–C, the microstructural characterization of CAs and CAs-Fe was initially conducted using SEM. Figure 1A illustrates the presence of numerous pores and a complex interconnected three-dimensional network structure on the surface of CAs. Figure 1B,C show SEM images of the CAs-Fe-700 and CAs-Fe-1000, respectively. It is observable that the CAs-Fe-1000 exhibit a higher density of pores and a significantly denser network structure compared with the CAs-Fe-700. This observation could be attributed to the higher carbonization temperature, leading to a reduction in material volume, thus forming a denser three-dimensional network structure. This structure increases the material’s specific surface area, ensuring more contact area between the material and the analyte, thereby enhancing the material’s electrocatalytic performance [27]. Additionally, the SEM images did not reveal the presence of iron nanoparticles, which could be due to the encapsulation of iron nanoparticles by carbon or the formation of the FeC structure.

The crystal structure of CAs was determined using XRD. As shown in Figure 1D, the peak observed at 23° in the XRD pattern of CAs can be attributed to the (111) plane of graphene carbon [28]. The XRD pattern of the CAs-Fe-700 and CAs-Fe-1000 indicate the presence of α-Fe, with a diffraction peak at 44.8°, and FeC, with obvious diffraction peaks at 38.8°, 42.8°, 43.5°, 44.6°, 46°, 48°, and 49.2°, in both samples [29]. Comparatively, FeC is more pronounced in the CAs-Fe-1000, while the Fe peak is weaker, possibly due to the higher carbonization temperature, leading to the conversion of Fe into FeC [30]. Furthermore, the graphene carbon peak observed at 26° in the CAs-Fe-700 is lower and wider, indicating a lower degree of graphitization, possibly due to the lower temperature during the carbonization process.

**Figure 2:**
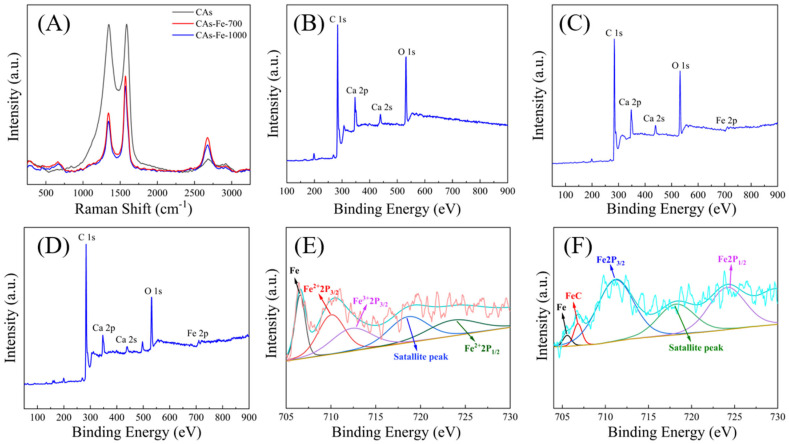
(**A**) Raman spectra of CAs, CAs-Fe-700, and CAs-Fe-1000. (**B**) XPS spectrum of CAs. (**C**) XPS spectrum of CAs-Fe-700. (**D**) XPS spectrum of CAs-Fe-1000. (**E**) Fe 2p for CAs-Fe-700. (**F**) Fe 2p for CAs-Fe-1000.

In the Raman spectra of materials (Figure 2A), the peaks corresponding to the D band and G band are centered at 1350 and 1590 cm^−1^, respectively. The D band is associated with edge defects and highly disordered carbon [31], while the G band corresponds to the stretching of C=C bonds in the main chain and highly ordered carbon [32]. The intensity ratio of the D band to the G band, denoted as *R* (*I_D_*/*I_G_*), is often used to measure the degree of graphitization and the level of structural disorder in materials [33]. For CAs, *R* = 1.01 (*R* = *I_D_*/*I_G_*). In contrast, the R values for the CAs-Fe-700 (*R* = 0.62) and CAs-Fe-1000 (*R* = 0.59) are significantly lower than those of CAs (*R* = 1.01). The decrease in the *R* values for both materials may be attributed to the formation of FeC crystals, which can significantly enhance the material’s conductivity [34].

The XPS confirmed the composition and chemical states of the CAs, CAs-Fe-700, and CAs-Fe-1000. Figure 2B–D, respectively, show the elemental composition of the CAs, CAs-Fe-700, and CAs-Fe-1000. The elemental scans reveal the coexistence of C, O, and Ca elements in all three samples. In the CAs-Fe-700 (Figure 2C) and CAs-Fe-1000 (Figure 2D), the presence of the Fe 2p peak at 712 eV indicates the presence of iron elements in the samples. Upon calculation, the C:O ratio in the CAs is 6.56, while it is 5.32 for the CAs-Fe-700 and 6.90 for the CAs-Fe-1000. This is attributed to the higher degree of graphitization of the material at higher temperatures, leading to a relatively higher carbon content, consistent with the previous XRD characterization results. The Fe 2p spectra of the CAs-Fe-700 and CAs-Fe-1000 are shown in Figure 2E,F, respectively. Besides the peaks corresponding to 2p^3/2^ and 2p^1/2^, a characteristic peak of metallic Fe is detected at 706.5 eV in Figure 2E [35]. The peaks observed at 706.1 and 707.2 eV in Figure 2F correspond to metallic Fe and FeC [36], respectively. It can be verified that both metallic Fe and FeC coexist in the CAs-Fe-1000, while no separate FeC peak is observed in the Fe 2p spectrum of the CAs-Fe-700, possibly due to the lower content of FeC in the CAs-Fe-700. Comparatively, the Fe peak in the CAs-Fe-1000 is significantly lower than that in the CAs-Fe-700, possibly due to surface oxidation of iron nanoparticles and the formation of FeC [37], consistent with the XRD characterization results.

### 3.2. Electrochemical Detection of DA

**Figure 3:**
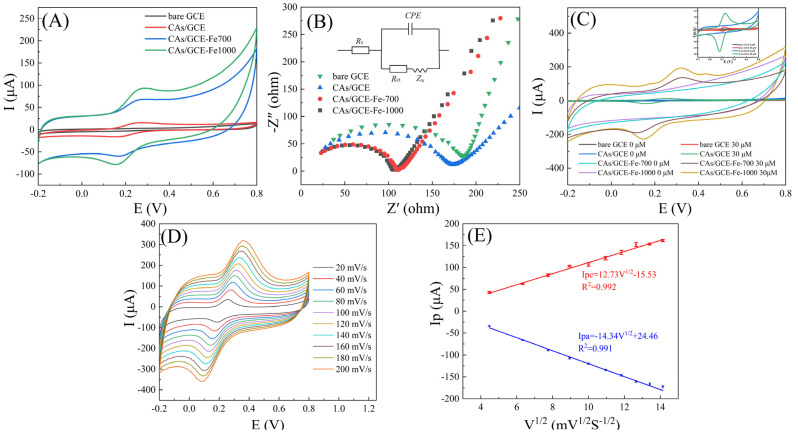
(**A**) CVs of various electrodes in 0.1 M KCl solution containing 1.0 mM of [Fe(CN)_6_]^3−/4−^. The CV was measured with a scan rate of 50 mV s^−1^. (**B**) The EIS of bare GCE, CAs/GCE, CAs/GCE-Fe-700, and CAs/GCE-Fe-1000. (**C**) CVs of bare GCE, CAs/GCE, CAs/GCE-Fe-700, and CAs/GCE-Fe-1000 in the absence/presence of 30 μM DA in PBS (pH = 7.0). The scan rate is 50 mV s^−1^. (**D**) CVs of the CAs/GCE-Fe-1000 at different scan rates from 20 to 200 mV s^−1^ in 0.1 M PBS (pH = 7) containing 30 μM DA. (**E**) Plots of the redox peak current (Ip) vs. the square root of scan rate (V^1/2^).

To evaluate the electrochemical activity of the CAs/GCE, CV experiments were performed on the bare electrode (bare GCE) and the CAs/GCE-Fe. Figure 3A presents the CV curves of bare GCE, CAs/GCE, CAs/GCE-Fe-700, and CAs/GCE-Fe-1000 in a 0.1 M KCl solution containing [Fe(CN)_6_]^3−/4−^ (1 mM), respectively. All electrodes exhibit distinct oxidation-reduction peaks, with peak currents observed in the order of bare GCE < CAs/GCE < CAs/GCE-Fe-700 < CAs/GCE-Fe-1000. The value of the oxidation-ereduction peaks is shown in Table S1. This observation indicates that the electron transfer rate of the iron-doped modified electrode is higher than that of the undoped iron CAs/GCE and bare GCE. This suggests that CAs-Fe exhibit excellent conductivity, which may be attributed to the doping of Fe nanoparticles and the formation of FeC crystallization.

As shown in Figure 3B, EIS tests were performed to analyze the differences of interface properties and electrochemical impedance on various electrodes. According to the Nyquist diagram shown in Figure 3B, the CAs/GCE-Fe-1000 have the smallest charge transfer resistance (Rct) and the sequence is CAs/GCE-Fe-1000 < CAs/GCE-Fe-700 < CAs GCE < bare GCE. This indicates that the modification of the electrode promotes charge transfer and the CAs/GCE-Fe-1000 have a higher charge transfer rate [38]. Figure 3C illustrates the electrochemical responses of four different working electrodes (bare GCE, CAs/GCE, CAs/GCE-Fe-700, and CAs/GCE-Fe-1000) towards DA. CV measurements were conducted in 0.1 M PBS solution (pH 7.0), with a scan rate of 50 mV s^−1^. The inset in the top right corner shows an enlarged view of the CV measurements for the bare GCE and CAs/GCE. In the absence of DA, no oxidation-reduction peaks were observed for the four electrodes. Upon introduction of 30 μM DA, peak currents were observed in the order of bare GCE < CAs/GCE < CAs/GCE-Fe-700 < CAs/GCE-Fe-1000. The peak currents of various electrode are shown in Table S2. The bare GCE exhibited weak peaks, while the CAs/GCE displayed significant oxidation-reduction peaks, with peak currents significantly higher than those of the bare GCE. The peak current of the CAs/GCE was approximately eight times that of the bare GCE. In addition, the CAs/GCE-Fe-700 and CAs/GCE-Fe-1000 showed significantly higher peak currents compared to the former two, approximately 7–9 times that of the CAs/GCE. This indicates that the CAs/GCE-Fe exhibit excellent electrocatalytic activity for DA detection, making it more suitable for DA detection. This may be attributed to the addition of Fe, which improves the conductivity of the material and enhances the charge transfer rate, thereby improving the current response in the detection of DA [39].

To better understand the alternating oxidation kinetics on the CAs/GCE-Fe-1000, we used CV testing to study the effect of scan rate on the oxidation current. Figure 3D shows the CV response of the CAs/GCE-Fe-1000 towards DA (30 μM) in PBS solution (pH 7.0) at different scan rates (20–200 mV s^−1^). Three CAS/GCE-Fe-1000 were chosen for reproducibility tests in each scan rate With increasing scan rate, the oxidation peak potential (E_pa_) shifted towards the positive direction and the reduction peak potential (E_pc_) shifted towards the negative direction. Meanwhile, both oxidation current (I_pa_) and reduction current (I_pc_) increased.

Figure 3E shows the linear relationship between peak current (Ip) and the square root of scan rate (V^1/2^). The RSD value is shown in Table S3. The linear regression equation is as follows:

I_pc_ = 12.73V^1/2^ − 15.53 (R^2^ = 0.992) (1)

I_pa_ = −14.34V^1/2^ + 24.46 (R^2^ = 0.991) (2)

Based on Equations (1) and (2), it can be concluded that the oxidation and reduction in DA on the CAs/GCE-Fe follow a diffusion-controlled mechanism [40].

**Figure 4:**
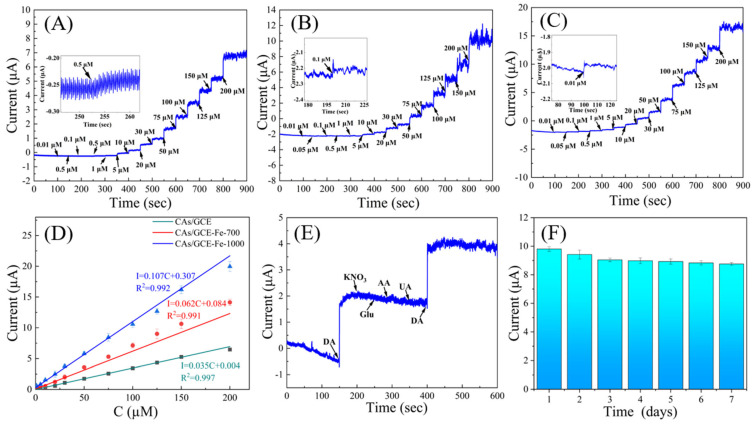
I-T response of (**A**) CAs/GCE, (**B**) CAs/GCE-Fe-700, and (**C**) CAs/GCE-Fe-1000 for various DA concentrations (0.01–200 μM) in 0.1 M PBS solution (pH = 7.0), with a scan rate of 50 mV s^−1^. (**D**) Linear calibration of I-t response with the DA concentration from 0.01 to 200 μM. (**E**) The effect of different interfering species on the I-t responses of DA of the CAs/GCE-Fe-1000. (**F**) Stability of the CAs/GCE-Fe-1000 electrochemical platform for 7 days.

Figure 4A–C show the current-time (I-t) response curves of the CAs/GCE, CAs/GCE-Fe-700, and CAs/GCE-Fe-1000 at different DA concentrations. We chose three CAs/GCE-Fe-1000 electrodes for reproducibility tests in each DA concentration. As the DA concentration increases from 0.01 μM to 200 μM, the current response becomes more pronounced. It can be observed from Figure 4A–C that the CAs/GCE, CAs/GCE-Fe-700, and CAs/GCE-Fe-1000 produce significant current signals at DA concentrations of 0.5 μM, 0.1 μM, and 0.01 μM, respectively. Particularly, the CAs/GCE-Fe-1000 exhibit the widest detectable range, with larger current fluctuations, indicating their higher detection capability and sensitivity.

Based on the corresponding linear regression results (Figure 4D), the regression equations for the CAs/GCE are determined as follows: I = 0.35C + 0.004 (R^2^ = 0.997). The calculated detection limit for CAs/GCE is approximately 0.166 μM, with a detection range of 0.5~200 μM. For the CAs/GCE-Fe-700, the regression equations are as follows: I_pa_ = 0.062C + 0.084 (R^2^ = 0.992), resulting in a detection limit of about 0.033 μM and a detection range of 0.1~200 μM. Lastly, for the CAs/GCE-Fe-1000, the regression equations are as follows: Ipa = 0.107C + 0.307 (R^2^ = 0.991), yielding a detection limit of approximately 0.0033 μM and a detection range of 0.01~200 μM. The RSD value is shown in Table S4. Clearly, through comparison, the CAs/GCE-Fe-1000 exhibit a broader detection range (0.01~200 μM), lower detection limit (0.0033 μM), and higher sensitivity (0.107 A/mol).

Under the same conditions, DA (30 μM) was simultaneously added with different interferents (100-fold KNO_3_, glucose, and equimolar concentrations of AA and UA). Figure 4E shows the selectivity test of the CAs/GCE-Fe in PBS solution, with the addition of KNO_3_, GLU, AA, and UA. Clearly, these four substances exhibit minimal fluctuations in the I-t curve, but the presence of DA causes significant fluctuations, indicating the excellent selectivity of the CAs/GCE-Fe-1000 electrode [41].

To verify the stability of the modified electrode, three CAs/GCE-Fe-1000 electrodes were sealed and stored at −2 °C for 7 days, with daily electrochemical tests for DA, at a concentration of 30 μM. In order to conduct reproducibility tests, three CAs/GCE-Fe-1000 were selected for testing on each day. The RSD value is shown in Table S5. The results in Figure 4F indicate that, even after continuous use for 3 days, the response current of the CAs/GCE-Fe-1000 to DA remained at 92.2% of the initial value, reaching 89.2% after 7 days. This demonstrates that the electrochemical detection capability of the CAs/GCE-Fe-1000 for DA remains relatively unchanged, indicating the high long-term stability of the modified electrode [42].

As shown in Table 1, compared to other carbon-modified electrodes, the CAs/GCE-Fe-1000 exhibit a lower detection limit for DA, indicating a higher sensitivity of the CAs/GCE-Fe-1000 electrode towards DA. Additionally, it also demonstrates a wider detection range compared to other modified electrodes.

The effectiveness of the CAs/GCE-Fe-1000 in real sample analysis was validated through the detection of DA in human urine. Urine samples were centrifuged at 8000 rpm for 10 min, followed by dilution with 0.1 M PBS solution (pH 7.0) at a ratio of 1:100. DA was determined using the standard addition method, with each sample analyzed in triplicate. Table 2 presents the recovery results for human urine samples, with recovery rates ranging from 97.3% to 103.3% and the relative standard deviation (RSD) being less than 5%. Therefore, the electrochemical method for DA detection in actual samples is deemed reliable.

### 3.3. Detection Mechanism of DA

**Scheme 2:**
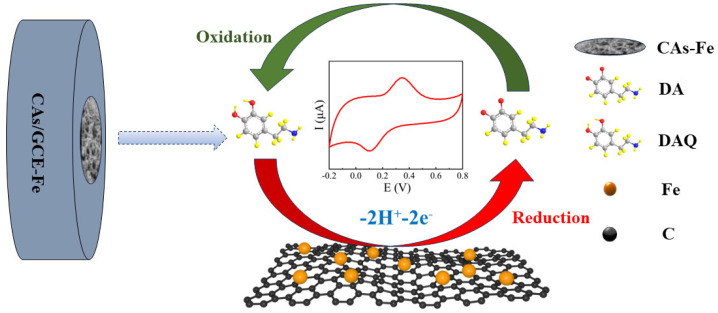
Illustration of the electro-oxidation process on the CAs/GCE-Fe surface towards DA.

As depicted in Scheme 2, the electrochemical detection of DA involves a catalytic process, in which DA tends to undergo oxidation by losing two electrons and two protons (2H^+^, 2e^−^) to form DA quinone (DAQ). The formed DAQ can also reversibly reduce back to DA, involving direct electron transfer in both oxidation and reduction reactions [51]. The presence of Fe and FeC metal crystals in the material not only enhances the conductivity, but also provides more active catalytic sites for electron transfer, significantly accelerating the electron transfer rate during the DA oxidation process. Additionally, as an electrode modification material, the CAs-Fe-1000 exhibit a superior electrocatalytic activity compared to the CAs-Fe-700. This could be attributed to the denser and more abundant network structure of the CAs-Fe-1000 and their higher degree of graphitization, providing favorable conditions for electron transfer in the electrocatalytic process.

## 4. Conclusions

In this study, we prepared a composite material, denoted as CAs-Fe, by introducing iron nanoparticles into CAs as a support, using AR and Fe(NO_3_)_3_ as raw sources. The CAs-Fe composite was utilized for the fabrication of an electrochemical DA biosensor. Through various characterization techniques such as Raman spectroscopy and XPS, we confirmed that the Fe doping occurred in the CAs and transformed from Fe to FeC with increasing temperature. The biosensor prepared with the CAs-Fe-1000 exhibited a high sensitivity (0.107 A/mol), a wide detection range (0.01~200 µM), and a low detection limit (0.0033 μM) in the electrochemical detection of DA. Furthermore, in the presence of various interferences such as GLU, uric acid, ascorbic acid, and urine, the CAs/GCE-Fe-1000 showed an excellent selectivity for DA. Satisfactory recovery rates were also observed in real sample testing. Based on the findings presented, we suggest that this composite CAs-Fe-1000 exhibits excellent electrocatalytic activity and can be effectively used for the electrochemical detection of DA.

## Figures and Tables

**Table 1:** Comparison of the sensing performance of the CAs/GCE-Fe-1000-based DA biosensor with other electrochemical DA biosensors.

Materials	Methods	Liner Range (µM)	Detection Limit (µM)	Ref.
GO/GCE	DPV	1.0–15	0.27	[43]
GME MWCNT/EPGCE	DPV DPV	4–100 1–20	2.64 0.23	[44,45]
GNPs-PANI/GCE	I-t	3–115	0.8	[46]
Au-Cu_2_O/rGO/GCE	DPV	10–90	3.9	[47]
K_2_Fe_4_O_7_-GCE	DPV	1–140	0.22	[48]
PABSA-rMOS2 2/CPE3	DPV	1–50	0.22	[49]
Au@Pt/GO/GCE	I-t	0.5–177.5	0.11	[50]
CAs/GCE-Fe-1000	I-t	0.01–200	0.0033	This work

**Table 2:** DA assay in human urine samples using the CAs/GCE-Fe-1000-based electrochemical biosensor.

Sample	Added (µM)	Founded (µM)	Recovery (%)	RSD (%)
Urine	0	ND	--	--
	1.5	1.46	97.3	3.84
	15	15.5	103.3	4.55
	30	29.7	99	3.77

## Data Availability

Data can be obtained upon request.

## Supplementary Materials

The following supporting information can be downloaded at: https://www.mdpi.com/article/10.3390/s24092787/s1. Table S1. The oxidation and reduction peak current of various electrodes in CV testing in 0.1 M KCl solution containing 1.0 mM of [Fe(CN)6]^3−/4−^. Table S2. The oxidation and reduction peak current of various electrodes in CV testing in the presence of 30 μM DA in PBS (pH = 7.0). Table S3. The RSD value of oxidation and reduction peak current under different scan rate(V^1/2^) for CAs/GCE-Fe-1000. Table S4. The RSD value of oxidation and reduction peak current under different DA concentration. Table S5. The RSD value of peak current in each day for CAs/GCE-Fe-1000.
