# Supplementary material for: Facile Synthesis of Fe-Doped, Algae Residue-Derived Carbon Aerogels for Electrochemical Dopamine Biosensors
Source: Sensors (Basel). 2024 Apr 27;24(9):2787. doi: 10.3390/s24092787 (PMC11086316; doi:10.3390/s24092787)
Supplement: Supplementary file 1 [file sensors-24-02787-s001.zip › sensors-2974825-supplementary.pdf]

SUPPLEMENTARY MATERIAL

Facile synthesis of Fe-doped algae residue-derived carbon aerogels for electrochemical dopamine biosensors

Hao Wu[1,#], Qin Wen[1,#], Xin Luan[2], Weiwei Yang[1], Lei Guo[1,*] and Gang Wei[2,*]

1 *Institute of Biomedical Engineering, College of Life Science, Qingdao University, Qingdao 266071, P.R. China*

2 *College of Chemistry and Chemical Engineering, Qingdao University, Qingdao 266071, P. R. China*

Correspondence: guoleibio@qdu.edu.cn (L.G.); weigroup@qdu.edu.cn (G.W.), Tel.: 0086-15066242101

SUPPLEMENTARY MATERIAL

Table S1. The oxidation and reduction peak current of various electrodes in CV testing in 0.1 M KCl solution containing 1.0 mM of $[Fe(CN)_6]^{3-/4-}$.

Electrode	Bare GCE	CAs/GCE	CAs/GCE-Fe-700	CAs/GCE-Fe-1000
Current-I_{pc} (μA)	0.681	23.03	27.04	56.78
Current-I_{pa} (μA)	-1.07	-22.92	-25.24	-64.12

Table S2. The oxidation and reduction peak current of various electrodes in CV testing in the presence of 30 μM DA in PBS (pH = 7.0).

Electrode	Bare GCE	CAs/GCE	CAs/GCE-Fe-700	CAs/GCE-Fe-1000
Current-I_{pc}(μA)	1.5	12.1	83.7	108.6
Current-I_{pa}(μA)	-1.1	-13.6	-94.5	-114

Table S3. The RSD value of oxidation and reduction peak current under different scan rate($V^{1/2}$) for CAs/GCE-Fe-1000.

Scan Rate ($V^{1/2}$)	RSD (I_{pc})%	RSD (I_{pa})%
4.47	2.408	1.620
6.32	1.291	1.704
7.75	3.012	1.342
8.94	2.003	2.079
10	3.911	2.082
10.95	3.921	2.030
11.83	4.726	2.707
12.65	4.509	2.525
13.42	2.203	3.717
14.14	2.563	3.403

SUPPLEMENTARY MATERIAL

Table S4. The RSD value of oxidation and reduction peak current under different DA concentration.

DA concentration (μM)	RSD of CAs (%)	RSD of CAs/GCE-Fe-700 (%)	RSD of CAs/GCE-Fe-1000 (%)
0.01	1.270×10^{-3}	1.528×10^{-2}	5.033×10^{-2}
0.1	4.160×10^{-3}	1.335×10^{-1}	2.517×10^{-2}
0.5	6.030×10^{-3}	1.307×10^{-1}	1.231×10^{-2}
1	1.234×10^{-2}	1.275×10^{-1}	1.528×10^{-2}
5	1.015×10^{-2}	4.163×10^{-2}	1.513×10^{-1}
10	2.101×10^{-2}	2.082×10^{-2}	4.041×10^{-2}
20	3.372×10^{-2}	2.517×10^{-2}	4.041×10^{-2}
30	5.132×10^{-2}	3.055×10^{-2}	4.940×10^{-1}
50	4.715×10^{-2}	3.753×10^{-1}	2.751×10^{-1}
75	2.347×10^{-1}	2.307×10^{-1}	2.843×10^{-1}
100	2.917×10^{-1}	2.646×10^{-1}	6.265×10^{-1}
125	4.258×10^{-1}	4.163×10^{-1}	5.346×10^{-1}
150	4.979×10^{-1}	8.008×10^{-1}	4.537×10^{-1}
200	8.737×10^{-2}	7.638×10^{-1}	6.331×10^{-1}

Table S5. The RSD value of peak current in each day for CAs/GCE-Fe-1000.

Day	RSD (%)
1	1.589×10^{-1}
2	3.129×10^{-1}
3	1.213×10^{-1}
4	1.993×10^{-1}
5	1.836×10^{-1}
6	1.520×10^{-1}
7	9.445×10^{-2}
